# Utilization of Human Induced Pluripotent Stem Cells for Cardiac Repair

**DOI:** 10.3389/fcell.2020.00036

**Published:** 2020-01-31

**Authors:** Chengming Fan, Eric Zhang, Jyotsna Joshi, Jinfu Yang, Jianyi Zhang, Wuqiang Zhu

**Affiliations:** ^1^Department of Biomedical Engineering, School of Medicine and School of Engineering, University of Alabama at Birmingham, Birmingham, AL, United States; ^2^Department of Cardiovascular Surgery, The Second Xiangya Hospital, Central South University, Changsha, China; ^3^Department of Cardiovascular Medicine, Physiology and Biomedical Engineering, Mayo Clinic, Scottsdale, AZ, United States

**Keywords:** exosomes, induced pluripotent stem cells, cardiac, repair, regeneration

## Abstract

The paracrine effect, mediated by chemical signals that induce a physiological response on neighboring cells in the same tissue, is an important regenerative mechanism for stem cell-based therapy. Exosomes are cell-secreted nanovesicles (50–120 nm) of endosomal origin, and have been demonstrated to be a major contributor to the observed stem cell-mediated paracrine effect in the cardiac repair process. Following cardiac injury, exosomes deriving from exogenous stem cells have been shown to regulate cell apoptosis, proliferation, angiogenesis, and fibrosis in the infarcted heart. Exosomes also play a crucial role in the intercellular communication between donor and recipient cells. Human induced pluripotent stem cells (hiPSCs) are promising cell sources for autologous cell therapy in regenerative medicine. Here, we review recent advances in the field of progenitor-cell derived, exosome-based cardiac repair, with special emphasis on exosomes derived from hiPSCs.

## Introduction

Cardiovascular disease caused by coronary obstruction accounts for up to 80% of all cardiovascular-related deaths (Lloyd-Jones et al., 2010). Cell-based therapies centered around the transplantation of stem cells and/or their derivatives into the infarcted heart, has been tested in preclinical and clinical studies over the past decade and demonstrated to be a promising strategy for the treatment of cardiovascular diseases (Barile et al., 2017b). Since the development of induced pluripotent stem cell (iPSC) technology in 2006 (Takahashi and Yamanaka, 2006), iPSCs have emerged as one of the most promising stem cell sources for therapeutic applications in cardiovascular field (Zhu et al., 2018). As patient-specific iPSCs are derived from patients themselves, they possess extensive self-renewal and differentiation potential. Despite encouraging advances in iPSCs technology, challenges remain in administration of iPSCs in the preclinic studies, including low rate of engraftment and the potential risk of tumorigenesis, which still impede clinical application of iPSCs and iPSCs-based derivatives (Jung et al., 2017; Taheri et al., 2019).

While the primary goal of stem cell-based therapy is to generate new cardiac muscle, recent data from both clinical and preclinical studies have indicated that transplanted stem cells may exert their functional beneficial effects largely through their secretome, by which the paracrine activity of the transplanted cells is mediated in part through their secreted vesicles (Merino-Gonzalez et al., 2016). Significant preclinical developments of exosome-based regeneration medicine have been achieved thus far through recognizing that it is the paracrine cues from transplanted iPSCs and/or its derivatives, that impart the major beneficial effects of regeneration within the injured tissues, rather than the direct effect of surviving transplanted cells (Wang et al., 2015; Dougherty et al., 2017, 2018; Ye et al., 2019). Furthermore, recent studies establish that exosomes derived by donor cells play a critical role in this paracrine regenerative mechanism (Kim et al., 2015; Merino-Gonzalez et al., 2016; Ju et al., 2018; Youn et al., 2019). In addition, significant functional improvement after intravenous cell therapy in patients with heart failure are most likely caused by the release of exosomal vesicles from the transplanted cells which are accumulated in organs such as the lung (Bartolucci et al., 2017). The application of stem cell−derived exosome-based technology as a cell-free strategy of preclinical application, has been identified in the progress of several fields, such as molecular diagnostic markers, drug delivery systems and potential target therapeutic agents (Joladarashi et al., 2015; Joladarashi and Krishnamurthy, 2017; Jung et al., 2017). In the cardiac regenerative medicine, stem cell-derived exosomes have been recognized as one of the key therapeutic factors, including the stimulation of cardiac repair of the injured heart tissues (Taheri et al., 2019). Exosomes are powerful mediators and functional regulators of cardiac cells, including cardiomyocytes and endothelial cells, which improve heart function and stimulate angiogenesis by enhancing regeneration of both blood vessels and injured myocardium in the peri-infarcted area; In general, the biologically active molecular cargoes include lipids, proteins and nucleic acids, such as DNA, mRNA, miRNA, and lncRNA. Thus, the potential advances of exosomes-based therapy include their role in the promotion of angiogenic, anti-apoptotic, anti-immunogenic, proliferative, or anti-fibrotic effects (Chaput and Thery, 2011; Wang et al., 2015; Yang, 2018; Ye et al., 2019). Therefore, exosomes indicate a huge therapeutic potential in the prevention and treatment of ischemic heart disease (Moghaddam et al., 2019).

In this review, we summarize the current advances on the utilizing exosomes to treat ischemic heart disease and conclude with a discussion of current challenges and future prospects in this field.

### Formation and Secretion of Exosomes

Extracellular vesicles (EVs) include exosomes (diameter range: 30–150 nm), microvesicles (diameter range: 50–1000 nm), and apoptosomes (diameter range: 50–5000 nm) (Barile et al., 2017b). Exosomes are secreted by most cell types (Kishore et al., 2016; Gao et al., 2017) and contain a variety of proteins and nucleotides (van der Pol et al., 2012). The secretion of exosomes is an ATP-dependent, multi-step process requiring transporter molecules (Colombo et al., 2014). In general, along with the inward budding of endosomal membrane, the primary exosomes are formed and then gradually mature with releasing into a structure known as multi-vesicular bodies (MVBs) (Taheri et al., 2019). After maturation, MVBs are either guided to destructive pathways or are secreted extracellularly by the cells (H Rashed et al., 2017). Cells use exosomes to deliver bioactive components for intercellular communication. For example, exosomes can enter into the target cells and deliver their cargo through a variety of endocytic pathways, including endocytosis, and clathrin-independent pathways, such as phagocytosis, macropinocytosis, caveolin-mediated uptake, and lipid raft mediated internalization (Mulcahy et al., 2014). Surface proteins on the exosome surface, including integrins, CD9, CD63 and CD81, are readily internalized by specific ligands in target cells and message delivery is mediated between cells via release of exosome cargo into the cytoplasm or nucleus of the recipient cells (Rana et al., 2012).

### Exosomes From Stem Cells

Exosomes equip a unique and powerful carrier for cells to deliver a variety of bioactive components and promote intercellular communication. Recently, exosomes from many stem cells, including mesenchymal stem cells (MSCs) (Lai et al., 2010), embryonic stem cells (ESCs) (Khan et al., 2015), cardiac progenitor cells (CPCs) (Barile et al., 2018), induced pluripotent stem cells (iPSCs) (Adamiak et al., 2018), and adipose-derived stem cells (ADSCs) (Xu et al., 2019) have been well investigated for their cardiac repair potency in several types of cardiovascular diseases, such as myocardial ischemia/reperfusion injury or myocardial infarction. Among these cell types, iPSCs is the most promising candidate for therapeutic applications because they can be generated from the patient’s own somatic cells, possess extensive cell-renewal capability and potentially provide a variety of cell types that can be re-administered to the patient (Zhu et al., 2018). On the other hand, contrary to the stem cell therapy, exosomes confer minimal tumorigenicity (Lai et al., 2015) and immune response (Bradley et al., 2002) as they are readily recognized and endocytosed by recipient cells or are metabolically eliminated through the blood and urine. Unlike iPSCs, the very limited number of endogenous MSCs, and the steadily dropping number of isolatable MSCs in patients, particularly in aging patients, make the therapeutic application of endogenous MSC-derived exosomes particularly challenging (Phinney and Pittenger, 2017). Resident CPCs in adult heart and their exosomes may be well suited to treat cardiac pathologies (Xiao et al., 2016). However, these exosomes are difficult to isolate in patients due to low availabilities. ADSCs are redundant and can be harvested from adipose tissues (Hong et al., 2019). ADSCs and their exosomes represent new approaches for myocardial repair (Perea-Gil et al., 2018; Bai et al., 2019; Yang et al., 2019). Cardiosphere-derived cells (CDCs) are cardiac progenitor cells with anti-inflammatory, anti-oxidant, anti-fibrotic, and cardiomyogenic properties (Gallet et al., 2017; Marban, 2018). ESC-derived exosomes may be obtained indefinitely from ESCs and large-scale of production is not a problem (Chen et al., 2019). However, the ethical issues about using ESCs in regenerative medicine remain as major challenges, in particular, human embryos are destroyed during the derivation of human embryonic stem cells (Pessina and Gribaldo, 2006). hiPSCs emerged as a promising alternative to human ESCs and have no ethic issues. hiPSCs-derived exosomes may be produced in large quantity, and are stable during cryostorage without loss of function. hiPSC are good cell resources for generating reliable “off-the-shelf” product and are ideal cell models for personalized medicine (Menasche, 2018). The advantages and disadvantages of exosomes from iPSCs and other stem cells were summarized in Table 1.

**TABLE 1:** Advantages and disadvantages of exosomes from different stem cells.

Origin of exosomes	Advantages	Disadvantages	References
MSC	Most studied, well isolated and purified	Low number of endogenous MSCs, and the constantly diminishing number of isolatable MSCs found in the aging individual	Phinney and Pittenger, 2017
CPC	CPCs are specialized to function in the heart, CPC derived exosomes may be particularly well suited to treat cardiac pathologies	Impractical to obtain a sufficient amount of CPCs from the limited amount of available heart tissue	Xiao et al., 2016
ADSC	Readily accessible by routine liposuction, higher number of stem cells can be harvested from adipose tissue	A consensus in the doses of exosomes has not been reached, and the related studies are inadequate and limited. The organ diseases that can be effectively treated by ADSC-Exos are limited.	Hong et al., 2019
CDC	Acellular and non-replicating, facilitating the development of a stable and reliable “off-the-shelf” product, less immunogenic	Low number of endogenous CDCs, need to isolated from the human heart	Gallet et al., 2017; Marban, 2018
ESC	Qualified exosomes can be obtained infinitely from ESCs; Capable of instigating cell analogous response in target cells.	Ethical issue: human embryos are destroyed during the process of harvesting embryonic cells, this makes the research unpopular with those that believe human life begins at conception and that this life is being destroyed	Pessina and Gribaldo, 2006; Chen et al., 2019
iPSC	iPSCs have emerged as a promising alternative to ESCs; readily accessible, possibilities of large-scale production, stability after cryostorage without loss of function and can be applied to personalized medicine.	Laborious and inefficient isolation techniques same as the isolation from other stem cells	Menasche, 2018
iCMs/iECs/iMSCs/iPgs	Generated from patient-specific iPSC-derivatives and can be used for an autologous therapy by activating endogenous repair.	Inefficient purity of iPSC-derivatives and exosomes isolation	Bian et al., 2019

*ADSC, adipose-derived stem cells; CDC, cardiosphere-derived cells; CPC, cardiac progenitor cells; ESC, embryonic stem cells; iPSC, induced pluripotent stem cell; iCMs, iPSC-derived cardiomyocytes; iECs, iPSC-derived endothelial cells; iMSCs, iPSC-derived mesenchymal stem cells; iPgs, iPSC-derived cardiovascular progenitors; MSC, mesenchymal stem cells.*

Preclinical studies have shown that exosomes can be used as an effective therapeutic strategy for a variety of diseases as they regulate a broad range of cellular behaviors and promote intercellular communications. Secreted exosomes may be collected from the cell culture medium via well-established protocols (Momen-Heravi et al., 2013; Witwer et al., 2013; Greening et al., 2015). Pilot studies from Sahoo et al. (2011) have shown that exosomes derived from human CD34 positive bone marrow-derived cells promote the survival, proliferation and angiogenic activity of endothelial cells. Studies have also shown that almost all exosomes have potential to increase the survival and proliferation of cardiac cells, attenuate ischemic injury, promote angiogenesis, and improve heart function in both small and large animal models (Khan et al., 2015; Gallet et al., 2017; Gao et al., 2017). Khan et al. (2015) reported that ESCs-derived exosomes, after being directly injected to infarcted mouse hearts, promote cardiac pro-angiogenesis and cardiomyocyte survival, improve cardiac function and reduce fibrosis after myocardial infarction. More recent papers confirmed the therapeutic effects of exosome in other types of myocardial injuries (Chen et al., 2019; Singla et al., 2019) and explored the potential mechanism underlying the beneficial effects (Lee et al., 2017). The therapeutic delivery of exosomes through the circulation system by using miRNAs mimics or synthetic exosomes as cargos and vehicles have been widely studied. Exosomes from CPCs (CPC-EXOs), ADSCs (ADSC-EXOs), MSCs (MSC-EXOs) or other stem cells have indicated positive results in the field of ischemic heart repair, including stimulation of angiogenesis and suppression of apoptosis (Xu et al., 2019). Because of the specific miRNA and cytokines in the cargo of CPC-EXOs, the therapeutic effect of CPC-EXOs is reported to be better than that of CPCs and MSC-EXOs (Barile et al., 2018; Xu et al., 2019). Hypoxia pretreatment of ADSC-EXOs and regulation of miRNAs in ADSC-EXOs including miR126-Spred1-ERK1/2-MAPK signaling pathway in angiogenesis, miR93-5p/TLR4 and Wnt/β-catenin signaling pathways in alleviated apoptosis both enhanced the therapeutic efficacy of acute myocardial infarction, suggesting the effectiveness of engineered exosomes as an alternative therapy for ischemic heart disease and regenerative medicine (Xu et al., 2019). As such, it appears that exosomes secreted from different cell types and under different conditions carry vast different varieties of bioactive molecular cargoes and subsequently provide different biomolecular effects (Li et al., 2013). The recent *in vivo* cardiac protective effect of the exosomes released by iPSCs and their derivatives is summarized in Table 2. However, further endeavors are warranted to investigate the candidate cell types as potent sources of exosomes for therapeutic approaches (Rezaie et al., 2019).

**TABLE 2:** Cardioprotective effects of exosomes secreted by iPSC and its derivatives.

Releasing source	Cargo	Administration route	Animal model	Biological effect	References
iPSC	A set of miRNAs and proteins	Intramyocardial, 48 h after reperfusion	Mouse, I/R	Improve LV function, promote angiogenesis, ameliorate apoptosis and hypertrophy; no effect on infarct size.	Adamiak et al., 2018
iPSC	miR-21 and miR-210	Intramyocardial, before reperfusion	Mouse, I/R	Ameliorate apoptosis through suppression of caspase 3/7 activation	Wang et al., 2015
iPSC-CM	A set of miRNAs and lncRNAs	Intramyocardial, cell injection	Rat, MI	Improve cardiac function	Lee et al., 2017
iPSC-CM	RNAs, peptides, and small molecules	–	–	Salvage the injured cardiomyocytes in the peri-infarct region against apoptosis, necrosis, inflammation, remodeling, and fibrosis	Yang, 2018
iPSC-Pg	Enriched genes for tissue reparative pathways	Intramyocardial, 3 weeks after MI	Mouse, MI	Improve cardiac function, no effect on infarct size, hypertrophy and vascular density.	El Harane et al., 2018
iPSC-CM, iPSC-MSC, iPSC-EC	–	Intramyocardial after MI	Mouse, MI	Improve LVEF and restore the function of the injured myocardium	Vaskova et al., 2018

*I/R, ischemic reperfusion injury; iPSC, induced pluripotent stem cell; iPSC-CM, iPSC-derived cardiomyocytes; iPSC-EC, iPSC-derived endothelial cells; iPSC-MSC, iPSC-derived mesenchymal stem cells iPSC-Pg, iPSC-derived cardiovascular progenitors; LVEF, Left ventricular ejection fraction; MI, Myocardial infarction.*

### Anti-apoptotic Effects of iPSC Exosomes

Transplanted iPSCs produce paracrine factors that enhance survival of native cells in the ischemic heart, however, the mechanisms remain largely unknown. It has been reported that exosomes, a cell-free component secreted from iPSCs, contain some specific miRNAs and cytokines that can provide robust cardiac protection and promotion of myocardial regeneration (Moghaddam et al., 2019). Another study has reported that exosomes secreted by murine cardiac fibroblasts-derived iPSCs exhibit enhanced cytoprotection against acute ischemia/reperfusion-induced cardiomyocyte apoptosis by reducing the activity of caspase 3/7 proteins in the ischemic myocardium (Wang et al., 2015). The anti-apoptotic effects may be explained by cardioprotective miRNAs inside the iPSCs-derived exosomes, such as miR-21 and miR-210 (Wang et al., 2015). Another miRNA carried by exosomes, miR-92a, was also shown to have cardioprotective effects via inhibition of apoptosis, promotion of angiogenesis, and reduction of fibrosis in injured hearts (Lee et al., 2017). Although detailed mechanisms of their anti-apoptotic effects are not fully deciphered, exosomes from iPSC-CMs has been shown to possess a powerful cardiac protective effect by preserving the mitochondrial membrane potential, decreasing the translocation of Bax to the mitochondria, delaying the mPTP opening time, and inhibiting caspase 3/7 protein activity under (hypoxic/ischemic) conditions [need citations]. These cardiac protective effects depend on the ERK1/2 and p38-MAPK signal pathway (Gartz et al., 2018). It has been found that programmed cell death 6 interacting protein, also known as Apoptosis-linked gene 2–interacting protein X (ALIX), is an endosomal sorting complex required for transport complex-associated protein. ALIX plays direct role in exosome biogenesis such as packing the cargos, aiding in their entry into vesicles, and regulating the formation of vesicles (Baietti et al., 2012; Hurley and Odorizzi, 2012) and promotion of cell degeneration, aging, and apoptosis (Chatellard-Causse et al., 2002). In addition, a recent *in vitro* and in aortic rings *ex vivo* study reported that exosomes from ALIX-overexpressing and ALIX-knockout hiPSCs provide stronger and weaker therapeutic benefits respectively, against cisplatin and oxidative damage in epithelial, epidermal, and endothelial cells (Sun et al., 2019). Furthermore, exosomes released by iPSC-derived MSCs alleviate hepatic ischemia reperfusion injury (I/R) possibly by decreasing oxidative stress, reducing inflammatory responses and inhibiting apoptosis. In addition, exosomes secreted by iPSC-derived MSCs promote the growth, proliferation, and migration of human dermal fibroblast by stimulating ERK1/2 (Kim et al., 2018). A recent study reported that after 7 weeks of peri-infarct injections, the best preservation of left ventricle function was found in the exosome (released by iPSC-derived cardiovascular progenitors) injected hearts compared to those injected with iPSC-CMs, iPSC-derived cardiovascular progenitors or PBS. The authors found that the exosomes were enriched with signaling cues crucial for pathways beneficial to chronic heart failure, such as enhanced metabolism, growth, survival, proliferation, angiogenesis, vasculogenesis, and reduced organismal morbidity and mortality (El Harane et al., 2018).

### Pro-angiogenic Activities of iPSC Exosomes

Angiogenesis is the formation of new blood vessels that helps to establish and support the normal structure and function of the cardiac tissues. Angiogenesis is defined as the migration, development and differentiation of endothelial cells to form new blood vessels (Kubis and Levy, 2003). Exosomes secreted by various cell types have been demonstrated to possess proangiogenic effects. For instance, exosomes isolated from MSCs and CPCs promote migration of endothelial cells (Vrijsen et al., 2010), while exosomes derived from human pericardial fluid have been shown to stimulate the proliferation of endothelial cells (Beltrami et al., 2017). Furthermore, exosomes secreted from CDCs have shown stimulation of angiogenesis in tube formation assays and have also shown enhancement of vessel density when locally delivered to chronic infarcted mouse hearts (Ibrahim et al., 2014). A very recent study demonstrated that exosomes released by immune response-free monkey autologous iPSCs provided enhanced wound healing through promotion of angiogenesis and cell viability of injured endothelial cells in the wounded regions (Lu et al., 2019). On the contrary, a study has reported that the effects of hiPSC-derived exosomes on normal human umbilical vascular endothelial cells (HUVECs) were minimal (Ding et al., 2018). However, under high glucose conditions, these exosomes were able to reduce senescence of endothelial cells, promote cell proliferation and enhance the formation of capillary-like structures (Ding et al., 2018). Vaskova et al. (2018) compared the reparative capacities of the exosomes secreted by iPSC-derived cardiomyocytes (iCMs), endothelial cells (iECs), and MSCs (iMSCs) and they found that iCM, iEC, and iMSC-exosomes possess the pleiotropic ability to generate a capillary network and improve the function of the damaged myocardium. A recent study has demonstrated that hiPSC-CMs-derived exosomes stimulate *in vitro* angiogenesis in several facets of tube formation, accompanying with increased expression of growth factors such as PDGFA, VEGF2A, and FGF2 in endothelial cells (Dougherty et al., 2018). Investigations have demonstrated that miRNA-199b play key role in iECs differentiation by modulating VEGF expression via targeting Notch signaling (Chen et al., 2015; Du et al., 2016). Another study indicated that exosomes derived by hiPS-ECs is enriched with miR-199b-5p that significantly promotes neovascularization via transcriptional upregulation of VEGFR2, regulated through Jagged1/Notch1 signaling pathway (Ye et al., 2019). It is documented that exosomes derived from iPS-MSCs significantly enhance angiogenesis (Qi et al., 2016), and promote the proliferation, migration and tube-forming abilities of endothelial cells *in vitro* (Hu et al., 2015; Zhang et al., 2015), via the activation of PI3K/Akt signaling pathway (Liu et al., 2017).

### Pro-cell Cycle Effects of iPSC Exosomes

Recent studies have demonstrated the beneficial effects of exosomes in enhancing the cell cycle activity in animal models of MI. For instance, exosomes secreted by CDCs were found to promote the proliferation of cardiomyocyte in mouse MI hearts (Ibrahim et al., 2014). Similarly, exosomes derived by iMSCs promoted the proliferation of human fibroblasts *in vitro* in a dose-dependent manner (Zhang et al., 2015) while iMSCs-derived exosomes enhanced the viability and cell cycle progression in human keratinocytes and human dermal fibroblasts (Kim et al., 2018). Ye et al. (2019) in one of their studies treated bovine aortic endothelial cells with 100 μg/ml of hiPSC-CM-derived exosomes and found a significant increase in cell proliferation when compared to control (no exosomes). Khan et al. (2015) reported that mouse hearts treated with ESC-derived exosomes promote myocyte proliferation by enhancing cardiomyocyte cycling, as evidenced by both BrdU+ (S-phase) and phosphorylated histone H3 (PH3+; M-phase) cardiomyocytes, at 28 days of infarction when compared to those treated with embryonic fibroblasts-derived exosomes orsaline. Similarly, ESC-derived exosomes also stimulated mRNA expression of cyclins A2, D1, D2, and E1 but suppressed the expression of cyclin inhibitors p16, p19, p21, and p53 at day 5 following myocardial infarction (Khan et al., 2015), indicating that iPSC-derived exosomes may activate cell cycle activity and promote cell proliferation.

### Teratogenic Potential of iPSC Exosomes

Teratoma is a benign tumor formed by cells from all three germ layers. Although iPSCs and their derivatives have been demonstrated to be effective for myocardial repair, the teratogenic risk of these cells remains a concern and limits the routine clinical uses of these cells (Ben-David and Benvenisty, 2011; Zhao et al., 2011). Several studies had shown that iPSCs indeed form teratoma in mice and non-human primates (Zhao et al., 2011; Hong et al., 2016; Lu et al., 2019). iPSC-derived exosomes may possess unique advantage in this regard. Although exosomes secreted from iPSCs and its derivatives were reported to enhance angiogenesis and cell cycle of cells in the host animal hearts (Khan et al., 2015; Adamiak et al., 2018), exosomes are non-proliferative and chromosome-free. Therefore, exosomes are not expected to form teratoma-like tumor masses cells (Lu et al., 2019). iPSC-derived exosomes represent a special cell-free system to regenerate the injured myocardium which will have significant applications in cardiac regenerative medicine (Ailawadi et al., 2015; Das and Halushka, 2015; Singla, 2016).

### Anti-fibrotic Effects of iPSC-Exosomes

Although the beneficial components in the exosomes secreted from iPSCs are not fully identified yet, studies have shown that iPSC culture medium have the capability to improve alveolar epithelial wound-healing and reduce lung fibrosis in a lung epithelial wound healing model (Gazdhar et al., 2014). Furthermore, it is reported that exosomes derived from macrophage contain miR-155 which reduces the proliferation and stimulates inflammation of fibroblast during cardiac injury (Wang et al., 2017). However, other previous studies have shown that exosomes secreted from iMSCs promote proliferation and migration of human fibroblasts and also increase their secretion of collagens and elastin (Zhang et al., 2015). Secretions of type I and type III collagens, elastin and their associated mRNA transcripts in the fibroblasts were increased in response to the treatment of exosomes from hiPSC-MSCs in a dose-dependent manner. These discrepancies may be explained by the different bioactive components in exosomes derived by different cell types or even from the same cell type but under different conditions. Further studies need to be done to address these issues.

It is noteworthy to mention that in some cases, depending on cell- type as well as genotypic and functional status of the cells, specific components in the exosomes may not be beneficial but rather, harmful to the cardiovascular system (Barile et al., 2017a). For example, the progression of heart failure may alter the miRNA cargos in cardiac-derived exosomes and suppress their regenerative activities (Qiao et al., 2019). As an individual miRNA transferred via exosomes from different cell types or in different status may elicit divergent biological responses, exosomes extracted from the serum of dilated cardiomyopathy patients were reported not only to cause dramatic pathological changes in gene expression in both neonatal rat cardiomyocytes and hiPSC-CMs *in vitro* but were also associated with the accelerated heart failure progression in patients (Jiang et al., 2017). Exosome content may be manipulated via genetic modification of the cells. It is reported that exosomes secreted from GATA-4 overexpressing stem cells highly expressed several anti-apoptotic miRNAs and displayed better myocardial repair potency in ischemic heart diseases (Yu et al., 2015). Yi et al. (2019) reported that exosomes from osteocalcin-overexpressed EPCs showed a beneficial effect by stimulating the endothelial OCN-GPRC6A signaling. An et al. (2019) reported that exosomes from ADSCs overexpressing miR-21 promote vascularization. Despite these beneficial effects, it is worthy to note that these genetic modifications of donor cells may also cause unwanted changes in exosomes. Accordingly, the effects and safety of modified exosomes should be evaluated sufficiently before their clinical applications (Yamashita et al., 2018). Application of hiPSCs with a mutant genotype should be very cautious unless they are first corrected into wild-type genotype. Thus, a thorough investigation of iPSC-derived exosomes to identify the beneficial (“good guys”) and potential harmful components (“bad guys”) should be performed prior to the routine clinical application of exosome-based therapy (Jung et al., 2017).

### Targeted Delivery of Exosomes to Injured Heart and Future Perspectives

**FIGURE 1:**
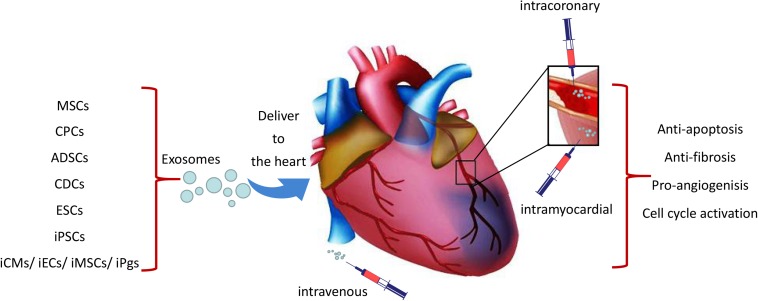
Schematic representation of the cardiac delivery of exosomes from iPSCs and other stem cells. Exosomes were delivered to the ischemic heart tissue through intravenous, intracoronary or intramyocardial which may lead to cardio-protection through the effects of anti-apoptosis, angiogenesis, anti-fibrosis and cell cycle activation.

Three different ways had been used to deliver exosomes to the ischemic heart tissue which include intravenous, intramyocardial and intracoronary injections (Figure 1). Exosomes signal cardiomyocytes and other supporting cells, including endothelial cells, smooth muscle cells and fibroblasts, and modulate their response to ischemic damage. Unfortunately, it is reported that majority of delivered exosomes are rapidly trapped in the liver, spleen, and lung when administrated intravenously, which may resulting in undesirable side effects and reduction of their beneficial pro-regenerative effects (Lai et al., 2014; Morishita et al., 2015; Smyth et al., 2015). Interestingly, when exosomes delivered 30 min after coronary occlusion and subsequent reperfusion, via an intramyocardial route led to significantly decreased infarct size in mini-pigs, and showed no effect through intracoronary delivery (Gallet et al., 2017). It is likely that exosomes injected via intracoronary route are prone to be flushed and drained through the coronary vein and subsequent uptake by macrophages, thereby resulting in poor retention in the heart (Barile et al., 2017b). As open chest surgery for intramyocardial injection of exosomes in MI patients is unlikely a viable approach from a translational perspective, there remains a need to maximize the therapeutic effects of exosome-based therapy. Targeted delivery of exosomes to the damaged cardiac tissue has emerged as an important goal in this field (Huang et al., 2019). Different attempts have been investigated to aid the targeted delivery of exosomes to the injured myocardium, such as fusion of biocompatible materials onto the stem cell surface membranes or modification of cell membrane via expression of cardiac or endothelial cell-specific surfaces markers (Li et al., 2018; Tang et al., 2018; Huang et al., 2019; Shen et al., 2019). In this perspective, Liu et al. (2018) generated an engineered hydrogel patch for prolonged release of extracellular vesicles secreted by hiPSC-CMs. They demonstrated that the delivery of this engineered hydrogel reduces infarct size, cardiomyocyte apoptosis and hypertrophy, and improve heart function in a rat MI model. Likewise, Vandergriff and colleagues chemically marked and ameliorated exosomes with a cardiac homing peptide (CHP; CSTSMLKAC) in order to augment their accumulation in the peri-infarct site after intravenous administration. Such targeted delivery has been shown to increase the myocardial reparative capacity of exosomes (Vandergriff et al., 2018) and has established the intravenous administration of exosomes as a feasible approach for myocardial repair.

**FIGURE 2:**
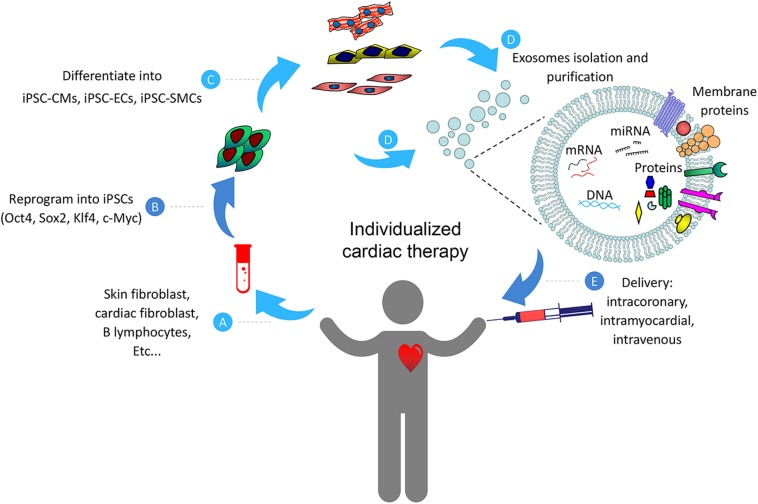
Schematic representation of the individualized cardiac therapy with exosomes generated from iPSCs and its derivatives. Patient-specific Cells **(A)** were isolated and reprogrammed into iPSCs **(B)**, then differentiated into its derivatives including iPSC-CMs, iPSC-ECs and iPSC-SMCs **(C)**. Exosomes **(D)** generated from iPSCs and its derivatives were then used as a platform for personalized cardiac therapy by simulating endogenous repair through intracoronary, intramyocardial or intravenous delivery **(E)**.

Although detailed mechanisms on exosome-based myocardial repair have not been fully understood, here we would like to summarize the potential beneficial effects of such therapy from current studies discussed in this review (Figure 2). Collectively, data from these studies indicate that naïve exosomes from iPSCs and/or their engineered vesicles (cell-free) may be effective for the treating heart diseases. However, despite the encouraging advances in this field at present, there are still number of challenges that need to be addressed before clinical promotion of exosome-based therapy. First, since exosomes from various types of stem cells may confer completely different response in target cells (Xiao et al., 2016), detailed investigations are necessary to identify the optimal cell types and determine the optimal functional status of these cells, and examine the molecular contents responsible for any observed beneficial effects of these exosomes. Second, the mechanism of exosome biogenesis, metabolic kinetics, curative effect, and *in vivo* biodistribution need to be carefully investigated (Taheri et al., 2019). Third, the number of exosomes stay in the cardiac area is very limited after being administered intravenously. This limitation has been shown to attenuate the therapeutic efficiency of the exosomes (Ni et al., 2019). Exosome-based cardiac therapies may be modified with the utilization of engineered biomaterials which provide platforms for durable retention and prolonged release of cargos in the injured myocardium.

## Author Contributions

CF, JZ, and WZ wrote the manuscript. EZ, JJ, and JY made the revision.

## Conflict of Interest

The authors declare that the research was conducted in the absence of any commercial or financial relationships that could be construed as a potential conflict of interest.
